# Behaviour of a New Composite Mesh for the Repair of Full-Thickness Abdominal Wall Defects in a Rabbit Model

**DOI:** 10.1371/journal.pone.0080647

**Published:** 2013-11-13

**Authors:** Gemma Pascual, Sandra Sotomayor, Marta Rodríguez, Yves Bayon, Juan M. Bellón

**Affiliations:** 1 Department of Surgery and Medical Specialties. Networking Research Center on Bioengineering, Biomaterials and Nanomedicine (CIBER-BBN). Faculty of Medicine, Alcalá University, Alcalá de Henares, Madrid, Spain; 2 Covidien – Sofradim Production, Trévoux, France; University of Minho, Portugal

## Abstract

**Introduction:**

Composite biomaterials designed for the repair of abdominal wall defects are composed of a mesh component and a laminar barrier in contact with the visceral peritoneum. This study assesses the behaviour of a new composite mesh by comparing it with two latest-generation composites currently used in clinical practice.

**Methods:**

Defects (7x5cm) created in the anterior abdominal wall of New Zealand White rabbits were repaired using a polypropylene mesh and the composites: Physiomesh^TM^; Ventralight^TM^ and a new composite mesh with a three-dimensional macroporous polyester structure and an oxidized collagen/chitosan barrier. Animals were sacrificed on days 14 and 90 postimplant. Specimens were processed to determine host tissue incorporation, gene/protein expression of neo-collagens (RT-PCR/immunofluorescence), macrophage response (RAM-11-immunolabelling) and biomechanical resistance. On postoperative days 7/14, each animal was examined laparoscopically to quantify adhesions between the visceral peritoneum and implant.

**Results:**

The new composite mesh showed the lowest incidence of seroma in the short term. At each time point, the mesh surface covered with adhesions was greater in controls than composites. By day 14, the implants were fully infiltrated by a loose connective tissue that became denser over time. At 90 days, the peritoneal mesh surface was lined with a stable mesothelium. The new composite mesh induced more rapid tissue maturation than Physiomesh^TM^, giving rise to a neoformed tissue containing more type I collagen. In Ventralight^TM^ the macrophage reaction was intense and significantly greater than the other composites at both follow-up times. Tensile strengths were similar for each biomaterial.

**Conclusions:**

All composites showed optimal peritoneal behaviour, inducing good peritoneal regeneration and scarce postoperative adhesion formation. A greater foreign body reaction was observed for Ventralight^TM^. All composites induced good collagen deposition accompanied by optimal tensile strength. The three-dimensional macroporous structure of the new composite mesh may promote rapid tissue regeneration within the mesh.

## Introduction

 Hernia repair continues to be one of the most frequent general surgery procedures [1]. A hernia is the protrusion of internal organs through the cavity which normally contains them. In technical terms, the repair of a hernial defect in the abdominal wall using a biomaterial has become routine clinical practice. After the development of a laparotomy (surgical technique involving the incision in the abdominal wall), the incidence of incisional hernia remains high, with estimates of up to 20% [2]. Some full-thickness abdominal wall defects caused by an incisional hernia [3,4] or an invading tumour [5] require the use of a biomaterial that shows optimal behaviour at each implant-host tissue interface [6]. It is possible to achieve the tissue repair of these defects using conventional prosthetic materials. However, such prostheses, generally in the form of a macroporous mesh, induce the generation of a peritoneal interface that is far from optimal with inadequate mesothelial development [7]. Because of this, *composite* or “double-layer” prostheses have been introduced. Besides comprising a macroporous mesh, these composites have an added laminar, or sheet, component to promote good peritoneal behaviour.

Such a prosthetic material should show the features proposed by some authors for the ideal prosthesis [8], including good host tissue incorporation, good biomechanical behaviour and the induction of optimal peritoneal regeneration [9]. 

Prosthetic materials with such properties designed in the form of a composite have two main components: one, usually of a loose macroporous structure, targeted at achieving good host tissue incorporation, and a smooth laminar component, whose objective is to create a peritoneal interface that will prevent tissue adhesion formation. The two components are usually bound together using an acrylic glue, are heat sealed or even sutured. Hybrid type prosthetic materials should not be considered composites since they consist of a single mesh composed of two polymer compounds [10]. 

While conventional biomaterials such as polypropylene or polyester achieve good abdominal repair both in terms of tissue and biomechanical behaviour, when placed in contact with the visceral peritoneum these materials can lead to complications such as tissue adhesions or abdominal obstruction [11], mesh migration to hollow organs [12], or to more serious complications such as intestinal fistula [13,14]. To avoid these adverse events, composites consist of two biomaterials one of which acts as a non-absorbable barrier [15]. Given its proven good peritoneal behaviour, one of the most used barrier components of composites is polytetrafluoroethylene (PTFE) [16,17]. Newer barrier materials for composites are absorbable conferring the advantage that when this component has been biodegraded, a reduced amount of foreign material is left in the host. This type of laminar prosthetic component is thought to have the benefit that any visceral tissue attachments formed soon after implant of the composite should in theory disappear as it becomes reabsorbed giving rise to an optimal peritoneal interface [18]. In general, whether reabsorbable or not, barriers should elicit a minimal inflammatory reaction and allow the rapid formation of a complete mesothelial lining to the prosthetic mesh [19]. 

When we pursue the full repair of the abdominal wall, we should not forget that a composite prosthesis, besides creating a good peritoneal interface, also needs to achieve the incorporation of host tissue in such a way that this confers compliance to the abdominal wall, which translates to patient comfort and good wall functionality [20]. 

 This study was designed to assess the behaviour of a new composite prosthetic mesh by comparing it with the two latest-generation composites currently used in clinical practice, each with a biodegradable barrier component. Through the use of sequential laparoscopy, special attention was paid to the changes produced at the peritoneal interface. The deposition of collagen induced by the different composites was also examined. This is important since the quality of the neoformed tissue at the implant site will in large measure determine the biomechanical properties of the repair zone [21]. 

## Materials and Methods

The study protocol adheres to the ARRIVE guidelines Kikenny, Plos one 2010, for the publication of animal studies [22].

The experimental animals used were 60 male New Zealand White rabbits weighing approximately 2500 g. The study was carried out in strict accordance with the Guide for the Care and Use of Laboratory Animals of the National and European Institutes of Health (Spanish law 32/2007, Spanish Royal Decree 1201/2005, European Directive 2010/63/UE and European Convention of the Council of Europe ETS123). All procedures were performed at the Animal Research Centre of Alcalá University. The study protocol was approved by the Committee on the Ethics of Animal Experiments of the University of Alcalá (registered code: ES280050001165).

### Biomaterials

**Figure 1:**
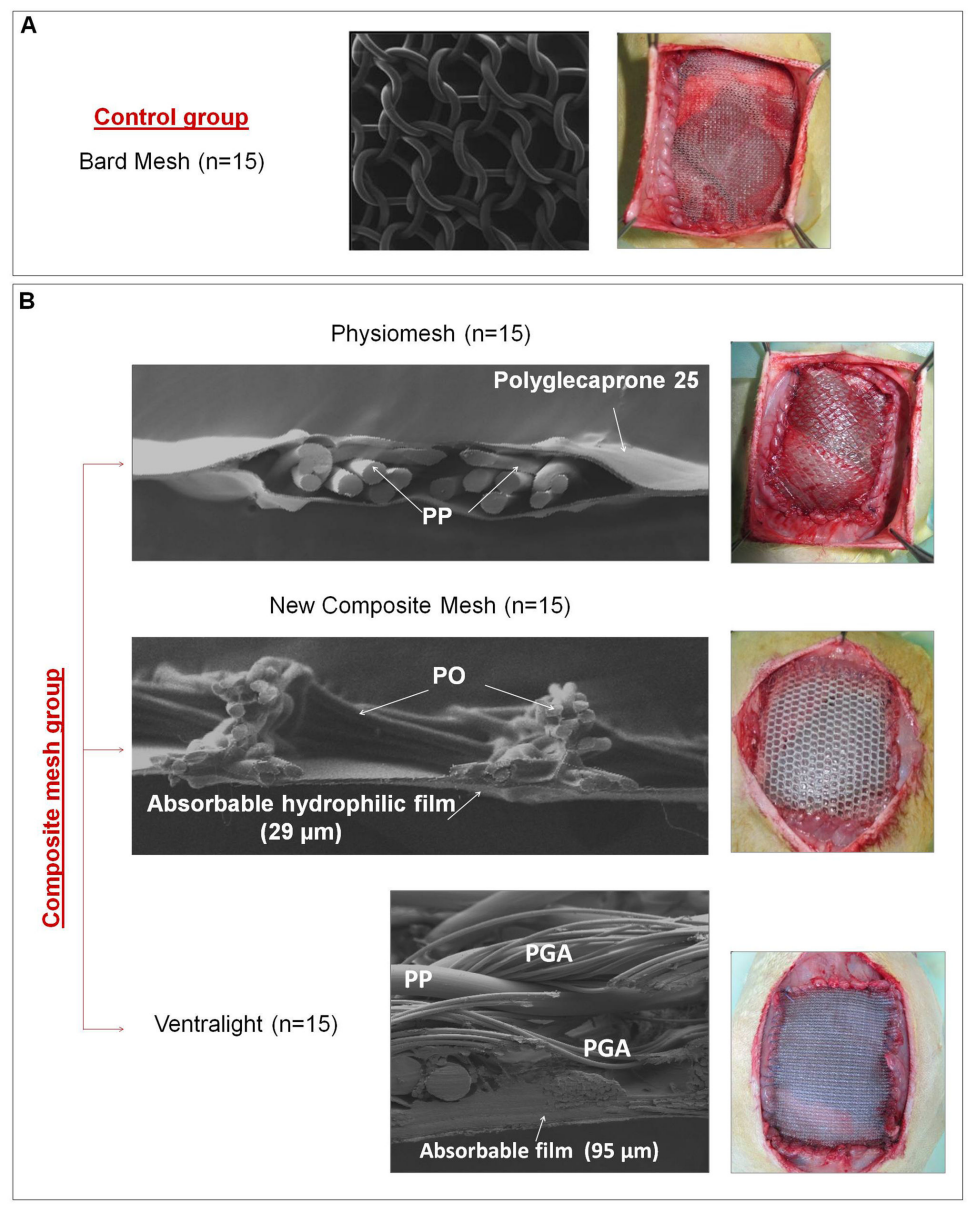
Characterization of the prosthetic materials. Scanning electron microscopy (SEM) images and macroscopic appearance of the meshes after their implant in the experimental animal. A) Control group: Bard mesh^TM^ (20x). B) Composites: Physiomesh^TM^ lateral view (20x); new composite mesh (20x) and Ventralight^TM^ lateral view (100x). Polypropylene (PP), Polyester (PO), polyglycolic acid (PGA).

The prosthetic implants used were (Figure 1): 

• *Bard^TM^* *Mesh* (Bard, Davol Inc., Warwick, RI, UK), a monofilament polypropylene (PP) mesh (control).
• *Physiomesh^TM^* (*Phy*) (Ethicon, Johnson & Johnson, Somerville, NJ, USA), a monofilament polypropylene mesh coated with a monocryl (polyglecaprone 25) layer on both its peritoneal and subcutaneous sides. A polydioxanone film binds the polyglecaprone 25 to the PP mesh.
• *Ventralight^TM^* (*Vent*) (Bard, Davol Inc., Warwick, RI, UK), a monofilament polypropylene mesh with a hydrogel barrier and bioreabsorbable polyglycolic acid (PGA) fibres. These fibres give strength to the barrier by binding it to the polypropylene mesh.
• New composite mesh (Ncm), a monofilament polyester 3D mesh with an absorbable layer of oxidized collagen and chitosan (Patent WO2009031047A2), on its peritoneal side. The quantity of collagen/chitosan is: oxidized collagen 95% / chitosan 5%. The mixture of polymers was casted as a 2.7% solution by weight of dry oxidized collagen 95%/chitosan 5% at a density of 0.133 g/cm^2^ (about 3.6 mg of dry oxidized collagen 95%/chitosan 5% per cm^2^; 3.4 mg oxidized collagen and 0.2 mg of chitosan). Chitosan is obtained by extensive deacetylation of the chitin. It is composed therefore of β(1-4) N-acetyl-2-amino-2-deoxy-D-glucose and 2-amino-2-deoxy-D-glucose units. The degree of acetylation (DA) is the percentage of β(1-4) N-acetyl-2-amino-2-deoxy-D-glucose over the total number of function. Selected chitosan is purified and reacetylated in order to obtain a DA 50% with random acetylation pattern. The source of the collagen is of porcine dermis origin. It was prepared according to the cited patent. In particular, it has been processed by a pepsin digestion step. Its purity is over 95% w/w and is 100% type I collagen without type III collagen and other major dermis proteins. Type I collagen is oxidized by periodic acid, generating aldehyde moieties from hydroxylysine residues. These aldehyde groups react with the free amine groups, provided by lysine residues and residual hydrolysine residues of collagen.

### Surgical technique

To minimize pain, all animals were administered 0.05 mg/kg buprenorphine (Buprecare^®^, Divasa Farmavic, Barcelona, Spain) 1 hour before and 3 days after the surgical procedure. Anaesthesia was induced with a mixture of ketamine chlorohydrate (Ketolar^®^, Parke-Davis, Spain), (70 mg/kg); diazepam (Valium^®^, Roche, Spain), (1.5 mg/kg); and chlorpromazine (Largactil^®^, Rhone-Poulenc, Spain), (1.5 mg/kg) administered intramuscularly. In some cases, an additional dose of anaesthetic was injected directly in the abdominal cavity during the course of surgery. 

Using a sterile surgical technique, 7x5 cm defects were created in the anterior abdominal wall comprising the aponeurotic, muscular and peritoneal planes. These defects were then repaired by securing the different prostheses to the edges of the defect with a running 4/0 polypropylene suture interrupted at the implant corners. The underside of the implants was placed in direct contact with the visceral peritoneum, while the upper side made contact with the subcutaneous tissue. The skin was then closed over the implants by running polypropylene 3/0 suture (Figure 1). 

Throughout the study, the animals were visually inspected for signs of dehiscence of the skin wound, seroma formation, wound infection and/or areas of mesh incompatibility. 

### Experimental design

Sixty animals were randomized to receive one of the four meshes: *Bard* (*n=15*, control group); *Phy* (*n=15*)*; Ncm* (*n=15*); and *Vent* (*n=15*)*.*

Following a protocol for experimental animal euthanasia in a CO_2_ chamber, 9 animals were sacrificed in each implant group at 14 days postsurgery and the remaining six at 90 days postsurgery. After sacrifice, a U-shaped incision was made in the abdominal wall to excise the mesh and some host tissue on both sides without disturbing adhesions. From the explanted meshes, fragments were obtained for light microscopy, scanning electron microscopy (SEM), biomechanical tests, quantitative reverse transcription polymerase chain reaction (qRT-PCR) and morphometric measurements. The remaining samples were stored at -80°C. Three animals per group were used for the tensile strength tests conducted at 14 days postimplant (these animals did not undergo laparoscopy). From each 7x5 cm implant in the abdominal wall, three 1.5 cm wide strips comprising the mesh and suture zones were obtained for these tests. 

### Laparoscopy study

On postoperative days 7 and 14, six animals per group were anaesthetized and examined laparoscopically to quantify adhesions between the visceral peritoneum and the implants. Laparoscopy was performed under general anaesthesia, introducing a Storz 3 mm, 0° laparoscope (Karl Storz, Tuttlingen, Germany) into the peritoneal cavity through a metal trocar (Karl Storz, Tuttlingen, Germany). Access was gained through the left lateral side of the abdominal wall. Pneumoperitoneum was achieved using CO_2_ at a maximum pressure of 8 mmHg. When the examination was completed, the laparoscopy equipment was removed and the skin closed. Observations were photographed and video recorded for subsequent review by two observers blinded to the identity of the mesh. 

The mesh surface area covered with adhesions was measured at each follow-up time. This was done by tracing the outlines of the adhesions on transparent polyethylene templates of the same size as the implants using the photographs taken during the laparoscopic study. At the end of the implant period (14 or 90 days), adhesions areas were again traced on the templates. Images obtained by digitalizing the templates were examined using image analysis software (*Image J. NIH;* http://www.rsbweb.nih.gov/ij/). 

Results are expressed as the percentage of implant covered by adhesions, ranging from 0% to 100% (no adhesions to completely covered). The intraabdominal structure involved, epiplon or intestine, location of adhesions and surface appearance of the implant were noted. 

Adhesions were classified according to their consistency as: a) loose, transparent and easily dissected; b) firm, whitish in colour and more difficult to dissect; or c) integrated within the prosthesis/visceral peritoneum interface and difficult to dissect away from the biomaterial and intestinal serosa [23]. 

### Morphological studies and collagen expression

Fragments of the implants plus surrounding host tissue were taken from the interfaces prosthesis/visceral peritoneum and prosthesis/subcutaneous tissue for morphological and immunohistochemical studies. Specimens were fixed in F13 fluid (60% ethanol, 20% methanol, 7% polyethylene glycol (PEG 300) and 13% distilled water), embedded in paraffin, and sliced into 5 µm-sections. Paraffin sections were deparaffinated in xylol and a graded alcohol series (100%, 96%, 70%), hydrated and equilibrated in PBS (phosphate buffered saline). For morphological analysis, sections were stained with haematoxylin-eosin and Masson’s trichrome and examined under a light microscope (Carl Zeiss, Oberkochen, Germany). 

To immunohistochemically label collagens, non-specific protein interactions were blocked using 3% BSA (bovine serum albumin) and specimens incubated with mouse monoclonal anti-collagen type I antibody, clone COL-I (C2456; Sigma, St. Louis, MO, USA) (1:400) and with mouse monoclonal antibody to type III collagen (hCL(III), clone III-53 (AF-5850; Medicorp, Montreal, Canada) (1:500). An immunofluorescence technique was used to detect the antigen-antibody reaction. A secondary antibody anti-mouse-rhodamine conjugated (715-295-150; Jackson Immunoresearch, Suffolk, UK) (1:300) was used for incubation in the dark for 1 hour at 37°C. Negative controls were incubated with 3% BSA instead of the primary antibody. Cell nuclei were counterstained with DAPI. The stained sections were examined under a Leica SP5 confocal microscope (Leica Microsystems, Wetzlar, Germany) to detect fluorescence. This work was performed by the Confocal Microscopy Service of the Universidad de Alcalá de Henares (UAH) and the Biomedical Networking Center (CIBER-BBN), located at the facilities of the Cell Culture Unit: www.uah.es/enlaces/investigacion.shtm.

For scanning electron microscopy, specimens were fixed in 3% glutaraldehyde placed in Millonig buffer, pH 7.3, and dehydrated in a graded acetone series. Critical point was reached in a critical point dryer (E-3000; Polaron, Newhaven, UK) with carbon dioxide. The pieces were then metalized with palladium gold and examined in a Zeiss scanning electron microscope (DSM-950). 

### Morphometric analysis of the peritoneum

The neoperitoneum formed over each implant was morphometrically assessed in 10 histological sections (in microscopy fields of magnification 10x) per group. In each tissue section, two random measurements were made of the thickness of the neoperitoneum, defined as the distance between the prosthetic material and the neoformed mesothelium. Images for analysis were captured using a digital camera fitted to a light microscope (Axiocam HR, Zeiss). The software used for these determinations was the Axiocam image analyzer (Axiocam HR, Zeiss). 

### Total RNA isolation and quantitative real-time PCR (qRT-PCR)

Tissue fragments (1 cm^2^ in size) were obtained from the implant area and stored at -80°C until use. RNA was extracted using guanidine-phenol-chloroform isothiocyanate extraction procedures with Trizol (Invitrogen, Carlsbad, CA, USA). RNA was recovered from the aqueous phase, precipitated with isopropanol, incubated overnight at -20°C and centrifuged several times with 70% ethanol. Amounts and purity were measured at an optical density of 260/280 nm and 260/230 nm in a NanoDrop ND-1000 spectrophotometer (Thermo Fisher Scientific Inc., DE, USA). 

Complementary DNA was synthesized from 200 ng of the total RNA by reverse transcription (RT) using oligo dT primers (Amersham, Fairfield, USA) and the M-MLV reverse transcriptase enzyme (Invitrogen). In parallel, a RT reaction was run without M-MLV to check the RNA sample lacked genomic DNA. 

Complementary DNAs were ampliﬁed using the following primers: collagen 1 (col 1) (sense 5´-GAT GCG TTC CAG TTC GAG TA-3´ and antisense 5´-GGT CTT CCG GTG GTC TTG TA-3´), collagen 3 (col 3) (sense 5´-TTA TAA ACC AAC CTC TTC CT-3´ and antisense 5´-TAT TAT AGC ACC ATT GAG AC-3´) and GAPDH (sense 5´-TCA CCA TCT TCC AGG AGC GA-3´ and antisense 5´-CAC AAT GCC GAA GTG GTC GT-3´). 

The RT-PCR mixture contained 5 μl of the inverse transcription product (cDNA) diluted 1:20, 10 μl of iQ SYBR Green Supermix (Bio-Rad Laboratories, Hercules, CA, USA) and 1 μl (6 μM) of each primer in a final reaction volume of 20 μl. RT-PCR was performed in a StepOnePlus Real-Time PCR System (Applied Biosystems, Foster City, California, USA). The samples were subjected to an initial stage of 10 minutes at 95°C. The conditions for cDNA amplification were as follows: 40 cycles of 95°C for 15 s, 60°C (collagen 1 and 3) or 55°C (GAPDH) for 30 s, and 72°C for 1 minute. UltraPureTM distilled water (DNase- and RNase-free) (Invitrogen) was used as a negative control in each reaction. 

The products were subjected to 2% agarose gel electrophoresis, stained with an SYBR Green II RNA gel stain (Invitrogen) and visualized with UV light. Gene expression was normalized against the expression value recorded for the constitutive gene glyceraldehyde 3-phosphate-dehydrogenase (GAPDH). 

### Macrophages

Paraffin–embedded tissues were immunolabelled using a monoclonal antibody against rabbit macrophages RAM-11 (DAKO M-633, USA) in the alkaline phosphatase-labelled avidin-biotin procedure. The method consists of the following steps: blockade with 3% BSA, incubation with the primary antibody (1:50 in PBS), incubation with immunoglobulin G (IgG) and biotin, and labelling with avidin. These steps were conducted at room temperature. The images were developed using a chromogenic substrate containing naphthol phosphate and fast red. Negative controls were incubated with 3% BSA instead of the primary antibody. Nuclei were contrasted for 5 minutes with acid haematoxylin. Labelled macrophages were quantified by performing counts in 10 microscopy fields (magnification x20) per sample under a Zeiss light microscope (Carl Zeiss, Oberkochen, Germany). Results were expressed as mean percentages of positive cells out of the total number of cell nuclei per section.

### Biomechanical tests

The tensile strength of the implanted biomaterials was measured using an INSTRON 3340 tensiometer equipped with pneumatic grips (Instron Corp., Norwood, MA, USA). Before testing, 1.5 cm-wide and approximately 2.5mm-thick (this parameter was exactly measured just before the test), strips comprising the mesh and suture zones were immersed in minimal essential medium (MEM) without fixation to avoid modifying the results. All measurements were made immediately after sacrifice. For all measurements, the interclamp distance was 7 cm and test speed was 2 cm/min. The ultimate tensile strength, expressed in Newtons (N), was analyzed in the study. The Load (N)/Elongation (mm) curves of the long term groups were also provided.

The Young's modulus of the different groups was also analyzed in the study.

### Statistical analysis

Adhesion percentages, collagen mRNA expression levels, macrophages and tensile strengths were expressed as means ± SEM and compared using the Mann Whitney U-test. All statistical analyses were performed using the GraphPad Prism 5 computer package for Windows. Significance was set at a probability (p) <0.05. 

## Results

### Macroscopy study

Hernia was detected in 3 out of the 15 animals receiving a *Phy* implant, in 2 of the 15 receiving *Ncm* and in one of the 15 receiving a *Bard mesh*. Hernias were detected in a macroscopic examination as a small tumouration at the implant margin. These hernias were confirmed by laparoscopy and then repaired by open surgery, in which their contents were reduced and the defect closed with a 4/0 polypropylene suture. No hernias were recorded for *Vent*. All hernias were detected early in the absence of infection signs by laparoscopy and could be repaired such that affected animals remained in the study. 

Seroma was evident at 14 days post-implant in 10/15 *Phy*, 6/15 *Ncm* and 13/15 *Vent* implants. In most cases, seroma appeared between the prosthesis and subcutaneous tissue. However, in 4 animals implanted with *Phy*, there were signs of seroma between the two polyglecaprone films coating the polypropylene mesh. We also detected seroma on the peritoneal-facing side of 4 *Vent* implants and 2 *Ncm*. No seroma was observed in Bard mesh group at 14 days. There were no cases of infection associated with seroma in any of the animals in the different groups. No seroma was observed in any of the groups at 90 days. 

### Laparoscopy & post-operative adhesion formation

At 7 days post-implant, the *Bard* mesh showed adhesions of the firm type affecting structures such as the omentum and intestinal loops. Only 2 specimens (1 in the 14-day group and 1 in the 90-day group) showed a reduced area occupied by adhesions on the lateral side of the implant. In both cases, omentum and viscera were involved. No differences in adhesions were observed at 14 days. At 90 days post-implant, adhesions were integrated within the prosthesis/visceral peritoneum interface and were difficult to dissect. The size of the affected area was similar to that observed at 14 days. 

Adhesions to the composite meshes at 7 and 14 days were loose or firm, mainly involved the omentum, were intensely vascularized and most of them only affected the upper third of the peritoneal implant side. For the *Phy* and *Ncm* implants, the intestinal loops were also involved, but for *Vent* only the omentum was adhered. At both time points, the absorbable component of *Phy* was clearly visible and no structural alterations could be macroscopically appreciated. However, loss of absorbable film continuity was observed for *Ncm* at 7 and 14 days post-implant. At 90 days post-implant, adhesions were now of a loose consistency and could be easily dissected. 

**Figure 2:**
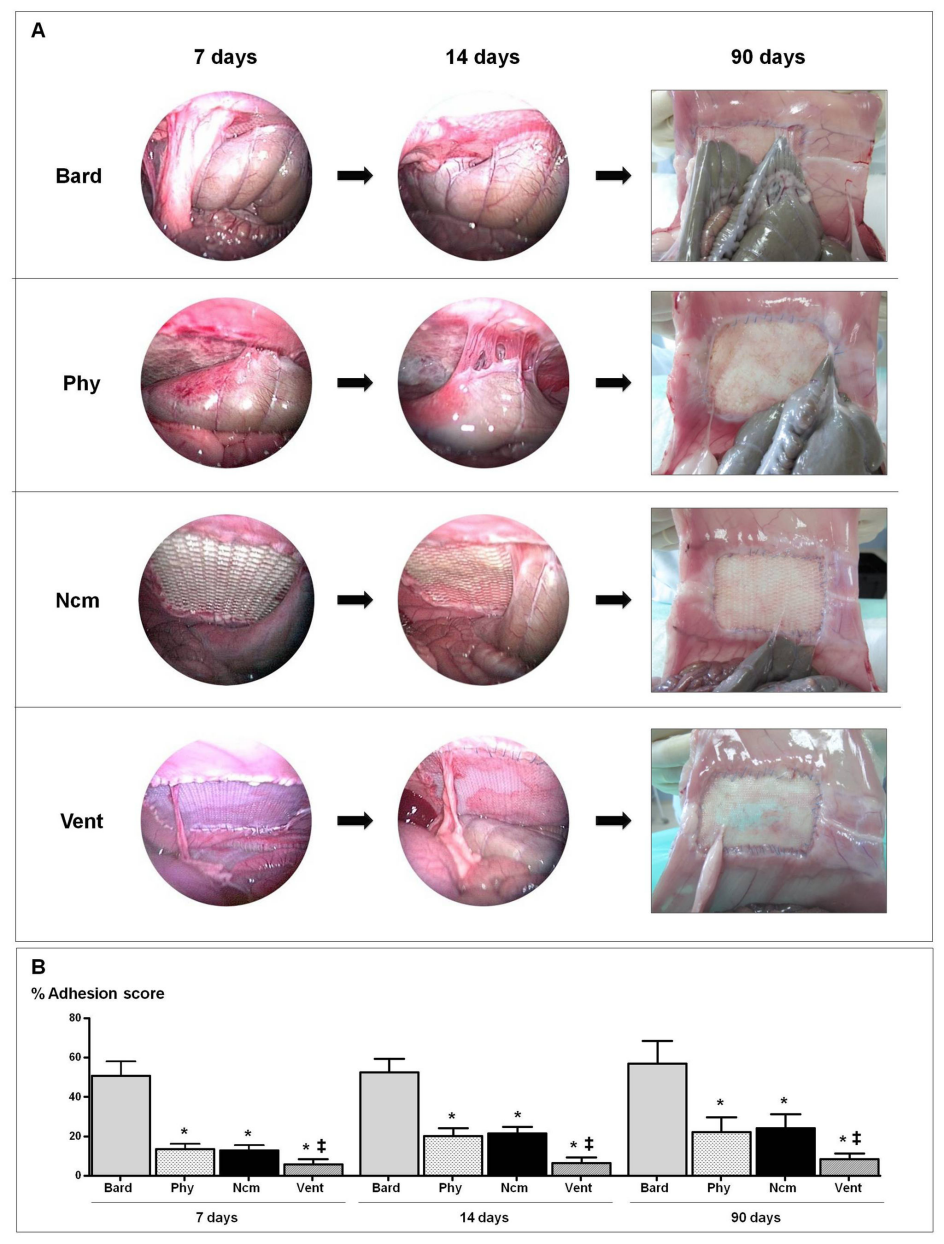
Laparoscopy study. A) Sequential laparoscopy tracking of adhesions 7, 14 and 90 days after implant in the groups: Bard mesh^TM^, Physiomesh^TM^, the composite mesh and Ventralight^TM^. B) Percentage prosthetic areas covered by tissue adhesions recorded for each group. Physiomesh^TM^ (Phy), new composite mesh (NCM), Ventralight^TM^ (VL). (*p<0.05 versus Bard; ‡p<0.05 versus Phy and NCM).

Our quantitative analysis of adhesions formation revealed that the macroporous mesh (*Bard*) induced a significantly higher adhesion percentage than the composites at each study time. Values recorded for *Phy* and *Nc*m were similar, without significant differences observed between them. The *Vent* implants showed the lowest percentages of adhesions, with significant differences observed compared to the other biomaterials. The trends recorded were similar at 7, 14 and 90 days for each mesh type. The severity of formed adhesions was without noticeable differences between the three composite meshes. Most adhesions were loose and easily dissected (Figure 2). 

### Morphological studies and collagen expression

Immunofluorescence confocal microscopy images were superimposed on differential interference contrast images (DIC) such that we could detect newly formed collagens (in red) on the translucent biomaterial. Nuclei were stained blue with DAPI. 

Fourteen days after implant, images of the macroporous *Bard* mesh revealed the presence of a loose neoformed connective tissue with extracellular matrix fibres running concentrically to the polypropylene filaments. For the composites, the filaments of the macroporous component were surrounded by a very similar loose connective tissue, while the fibres of the extracellular matrix of the tissue around the laminar component appeared to be more ordered and dense. The laminar absorbable component of *Phy* was visible at 14 days postimplant, and its reabsorption was complete at 90 days. The absorbable hydrophilic film of *Ncm* could not be detected at 14 days postimplant, and both at 14 and 90 days the imprint left by the mesh filaments could be seen on the neoperitoneum surface. For *Vent*, no remains of the hydrogel were observed at 14 days but its other absorbable component (PGA) could be seen, though these filaments showed signs of degradation. By 90 days postimplant, PGA filaments could no longer be observed. 

**Figure 3:**
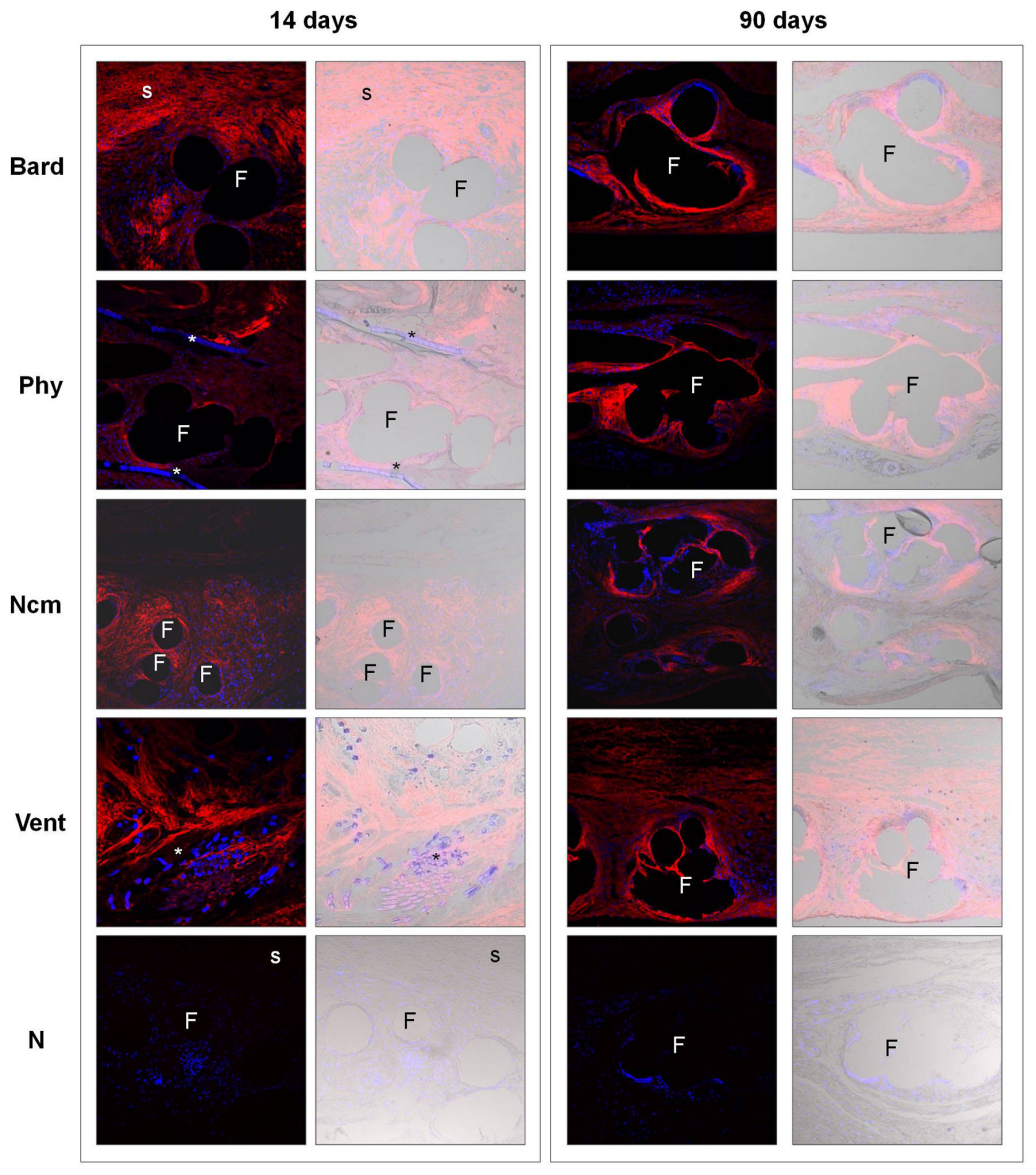
Collagen type I expression. Immunofluorescence detected for collagen type I at 14 and 90 days postimplant (left panel). N=negative controls; Bard mesh^TM^ (Bard) 14 days and Physiomesh^TM^ (Phy) 90 days. Neoformed collagen appears red and cell nuclei (stained with DAPI) appear blue. In the DIC images, the biomaterial appears translucent (right panel). Confocal light microscopy (200x). Subcutaneous tissue (s), prosthetic filaments (F), polyglycolic acid or polyglecaprone (*).

Collagen type I expression patterns were similar for all the implant groups. Most expression was seen around the prosthetic filaments, areas of newly formed tissue and subcutaneous tissue. *Phy* showed the faintest expression but a similar pattern. *Vent* showed the most intense expression at 14 days compared to the rest of the implants. In the long-term, the pattern of collagen type I expression was the same as at the previous time point though increases in staining and intensity were produced. In general collagen type I deposits were most abundant at 90 days postimplant in all the study groups (Figure 3). 

**Figure 4:**
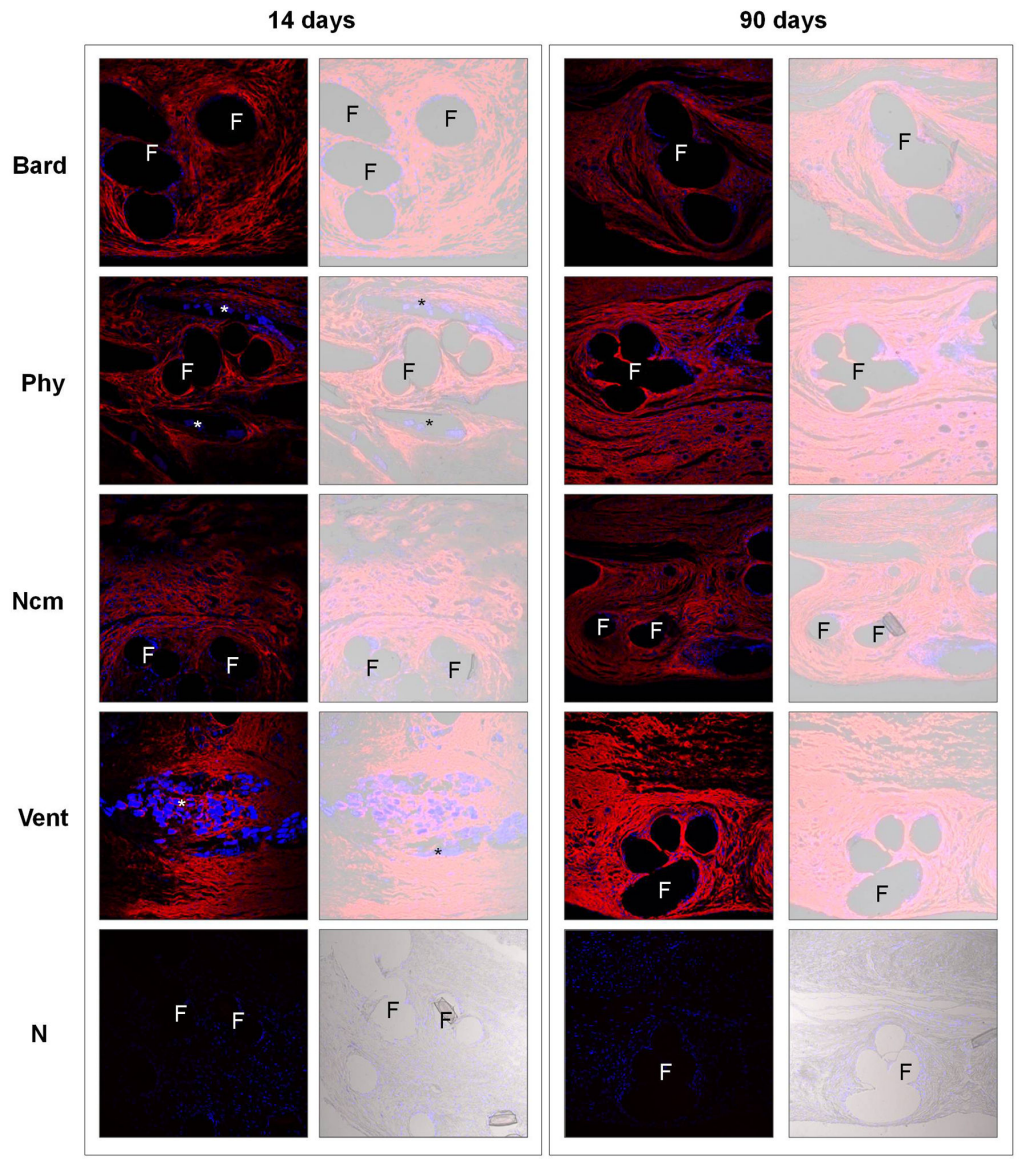
Collagen type III expression. Immunofluorescence detected for collagen type III at 14 and 90 days postimplant (left panel). N=negative controls; new composite mesh (NCM) 14 days and Ventralight^TM^ (VL) 90 days. Neoformed collagen appears red and cell nuclei (stained with DAPI) appear blue. In the DIC images, the biomaterial appears translucent (right panel). Confocal light microscopy (200x). Subcutaneous tissue (s), prosthetic filaments (F), polyglycolic acid or polyglecaprone (*).

Collagen III expression was homogeneously distributed in the neoformed tissue, around and between the different prosthetic components. The same pattern of expression was produced as at 14 days postimplant, but staining intensity was greater at 90 days for *Phy* and *Vent* compared to the earlier time point (Figure 4). 

### Morphometric analysis of the peritoneum

By day 14 postimplant, the *Bard mesh* showed a greater thickness of the neoperitoneum (164.45±34.79 μm) than the composites. No significant differences in this variable were observed among the *Phy* (128.22±28.42 μm), *Ncm* (121.32±25.14 μm) and *Vent* implants (129.49±17.51 μm). At 90 days, neoperitoneal thickness was similar for each of the biomaterials: *Bard mesh*, 96.43±24.76 μm; *Phy*, 98.65±11.82 μm; *Ncm* 73.89±9.04 μm and *Vent*, 92.90±6.47 μm.

### qRT-PCR

**Figure 5:**
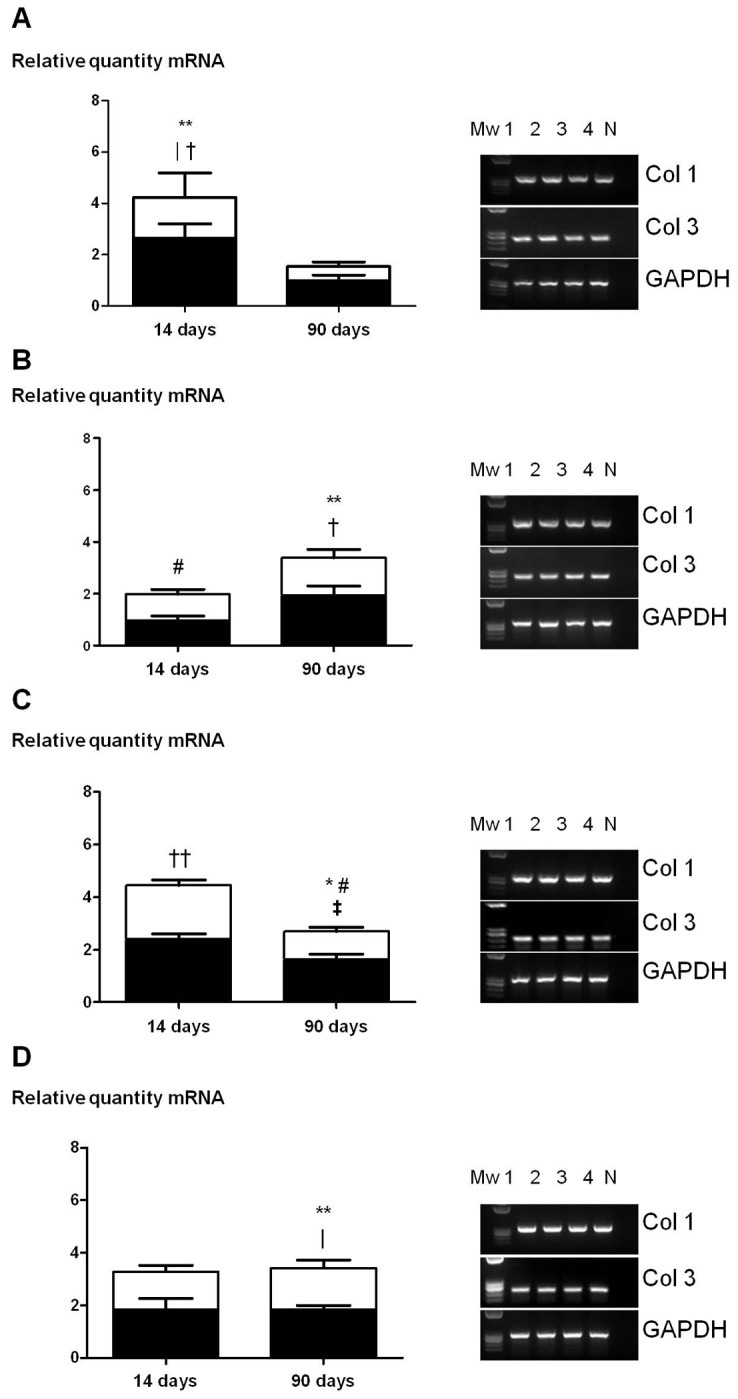
Collagen 1 and 3 mRNA expression determined by RT-PCR. Stacked bar charts of the relative mRNA amounts, collagen 1 (white bar) and 3 (black bar), recorded for Bard mesh^TM^ (A), Physiomesh^TM^ (B), the new composite mesh (C) and Ventralight^TM^ (D) at the different study times (left panel). Results are the mean ± SEM of three experiments. Gene expression was normalized to that of the GAPDH gene. Agarose gels obtained (right panel). Mw: molecular weight markers; 1/2: 14 days postimplant; 3/4: 90 days postimplant; N: negative. Col 1: *, p<0.05 and **, p<0.01 vs. Bard^TM^ at 90 days; #, p<0.01 vs. new composite mesh at 14 days. Col 3: |, p<0.05 vs. Bard^TM^ at 90 days; †, p<0.05 and ††, p<0.01 vs. Physiomesh^TM^ at 14 days; ‡, p<0.05 vs. new composite mesh at 14 days.

The stacked bar charts in Figure 5 show collagen 1 (white bars) and 3 (black bars) mRNA expression levels observed in the neo-tissue detected between the prosthetic filaments at both time points. In general, we observed a heterogeneous pattern of collagen 1 and 3 mRNA expression in the different types of prosthesis. The behaviour of *Bard* and *Ncm* mesh was very similar, with a maximum peak of expression for both types of collagen seen at 14 days that was significantly reduced at 90 days. *Phy* showed the opposite behaviour, with greatest expression recorded at 90 days, significantly so for collagen 3. Expression levels observed for *Vent* remained unchanged across the study time points. 

Collagen 1 mRNA levels detected for *Bard* mesh and *Ncm* fell significantly over time, showing relative reductions of 65% (p<0.01) and 47% (p<0.01), respectively. *Phy* and *Vent* showed similar collagen 1 mRNA expression. 

At 14 days postimplant, *Phy* exhibited the lowest collagen 1 expression compared to the remaining implants. For *Ncm*, collagen 1 mRNA expression (p<0.01) was two-fold that observed for *Phy*. *Bard* and *Vent* showed similar levels. At 90 days postimplant, significantly lower collagen 1 mRNA expression was detected for *Bard mesh* compared to *Phy*, *Ncm* and *Vent* (p<0.01, p<0.05 and p<0.01 respectively). *Vent* showed almost three times the expression noted for *Bard mesh*. No significant differences in collagen 1 mRNA expression levels were detected between *Phy*, *Ncm* and *Vent*. 

Collagen 3 mRNA levels recorded for *Bard mesh* and *Ncm* were significantly reduced between the two time points by 62% (p<0.05) and 32% (p<0.05) respectively, as occurred with collagen 1. Levels associated with *Phy* were double (p<0.05) those detected at 14 days. For *Vent*, collagen 3 mRNA levels were similar at both time points. 

At 14 days postimplant, *Phy* showed significantly lower collagen 3 mRNA expression than the levels recorded for *Ncm* and *Bard mesh* (p<0.01 and p<0.05 respectively) while highest expression levels were shown by *Bard mesh*. At 90 days post-implant, *Phy*, *Ncm* and *Vent* revealed similar collagen 3 mRNA expression, which was higher than that observed for *Bard mesh*. When comparing the three composites, expression levels were significantly higher only for *Vent* (p<0.05) compared to *Bard mesh*. 

### Macrophages

At 14 days postsurgery, control *Bard* specimens were moderately labelled with monoclonal antibodies against rabbit macrophages (RAM-11) and giant foreign-body reaction cells around the polypropylene filaments. At 90 days, the proportion of labelled cells had slightly dropped.

Two weeks after implant, the absorbable sheet of *Phy* showed signs of degradation and was surrounded by inflammatory cells and macrophages cells stained with the RAM-11 antibody. Polypropylene filaments were also surrounded by macrophages and giant foreign-body reaction cells. Despite the disappearance of the absorbable component at 90 days, a moderate number of macrophages were observed around the PP filaments.

In the *Ncm* specimens, RAM-11 positive cells showed a similar distribution pattern to that detected for the other mesh types, with macrophages and giant foreign-body cells observed around the polyester filaments at 14 and 90 days of implant.

For the *Vent* implants, the macrophage reaction was intense and significantly greater than that observed for the other composites at both follow-up times. The absorbable component (PGA) showed an intense inflammatory reaction at two weeks postimplant. Although this component was not detected at 90 days, the macrophage response remained intense in areas between the polypropylene filaments. 

**Figure 6:**
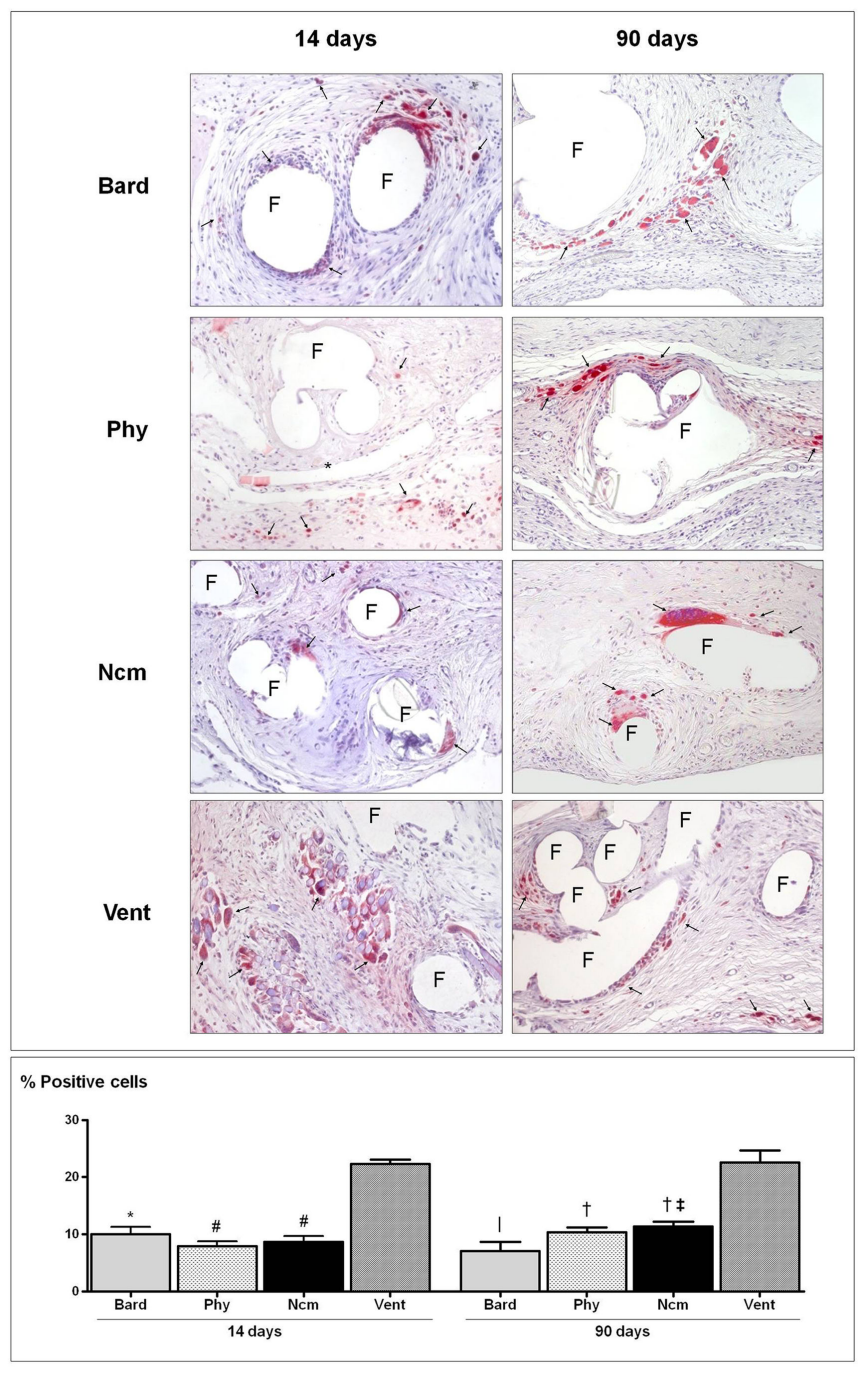
Foreign-body reaction of the different meshes. Immunohistochemical labelling of rabbit macrophages (arrows) using the RAM-11 monoclonal antibody (200x) (top panel). Positive cell percentages recorded after 14 and 90 days of implant (bottom panel). Results are expressed as the mean ± SEM. *, p<0.01 and #, p<0.001 vs. Ventralight^TM^ at 14 days; |, p<0.01 and †, p<0.001 vs. Ventralight^TM^ at 90 days; ‡, p<0.05 vs. Bard mesh^TM^ at 90 days. Prosthetic filaments (F), polyglecaprone film (*). Physiomesh^TM^ (Phy), new composite mesh (NCM), Ventralight^TM^ (VL).

In our study no significant differences in macrophage responses to each implant were recorded between the two study times, despite the complexity of the temporal changes in wound healing that will accelerate or delay tissue integration and therefore affect mechanical behaviour. At 14 and 90 days postimplant, the number of macrophages was significantly greater for *Vent* than for the other 3 implant groups (Figure 6). Thus, the macrophage pattern will vary with each implant, depending on the rate of degradation of the reabsorbable coatings.

### Biomechanical resistance

**Figure 7:**
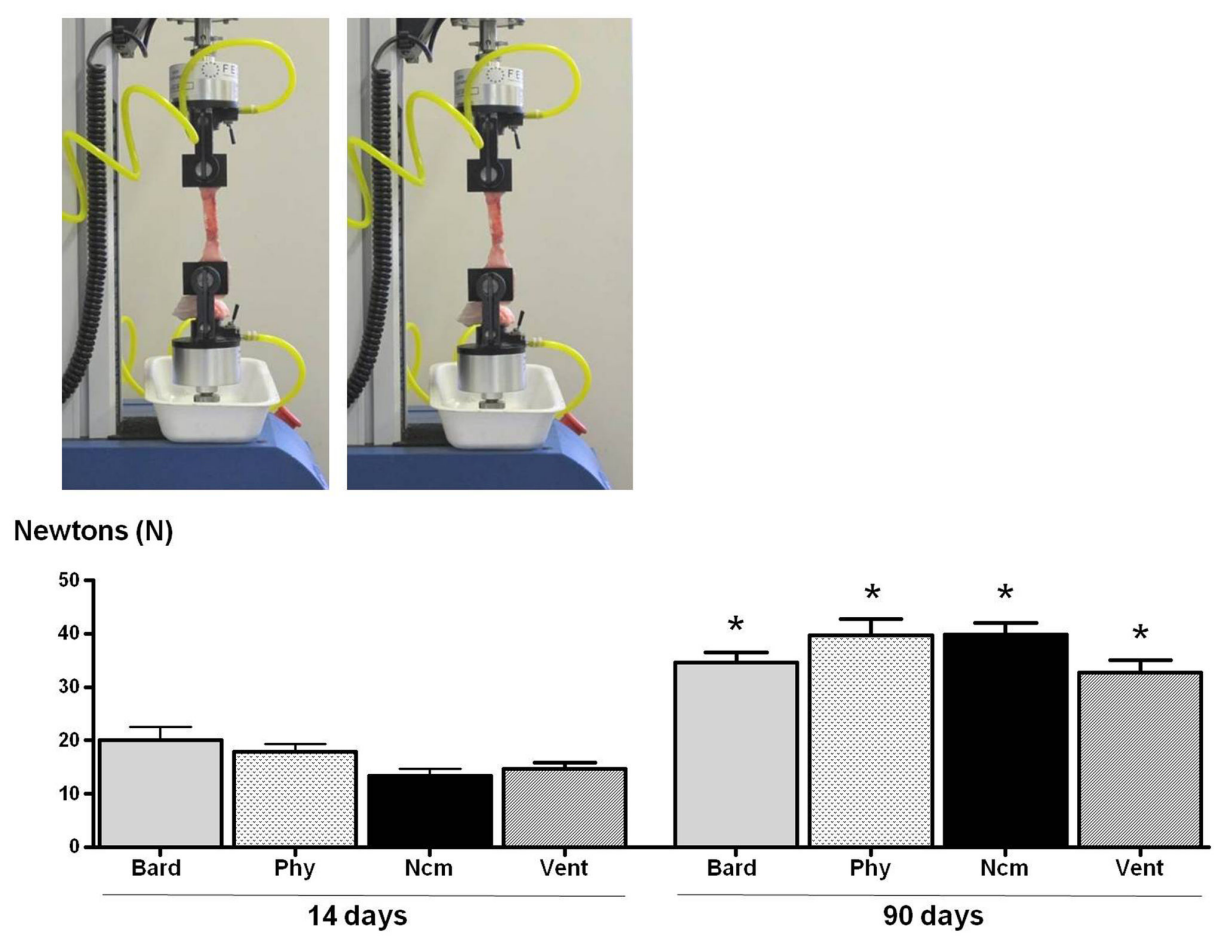
Biomechanical strength of the different meshes. Ultimate tensile strength of the different groups at 14 and 90 days postimplant. Bard mesh^TM^ (Bard), Physiomesh^TM^ (Phy), new composite mesh (NCM), Ventralight^TM^ (VL). (*p<0.05 versus 14 days).

Tensile strengths (expressed in Newtons) were similar for the different biomaterials at 14 days postimplant. By 90 days, tensiometry data obtained for the repair zone were similar for each prosthesis. In each implant group, significantly higher tensile strengths were recorded at 90 days versus 14 days (Figure 7). 

**Figure 8:**
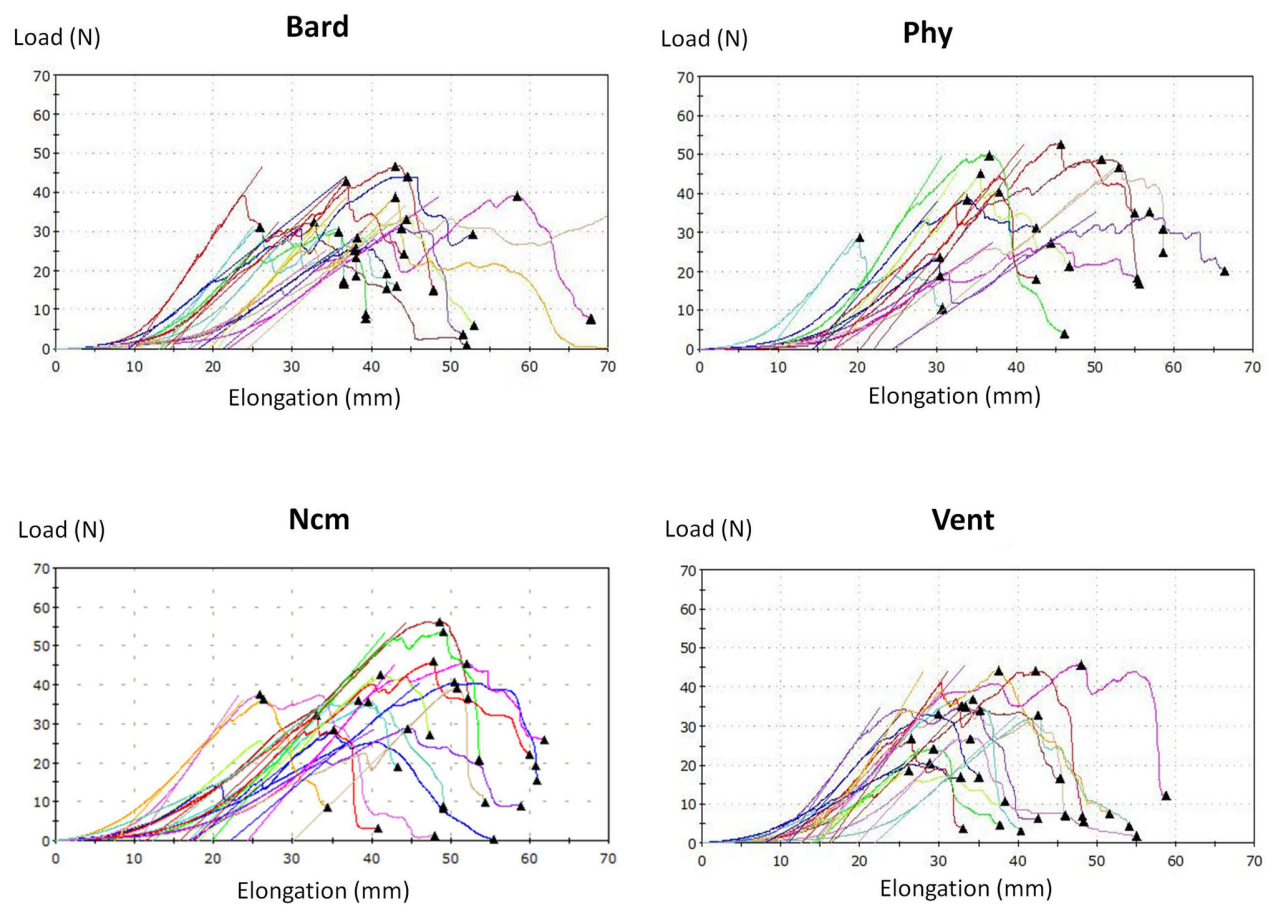
Load (N)/Elongation (mm) curves of the different meshes. The Load/Elongation curves of all the different groups, at 90 days, were represented in the figure. From these curves the Young´s modulus of the different groups of our study were calculated and statistically analyzed. Bard mesh^TM^ (Bard), Physiomesh^TM^ (Phy), new composite mesh (NCM), Ventralight^TM^ (VL).

Load/Elongation curves at 90 days were also showed in the Figure 8. As can be seen in the figure, in all the samples the stress and strain initially increase with a linear relationship, which is the linear-elastic portion of the curve. In this area of the curve, when the stress is reduced, the material is able to return to the original shape. The slope of the line in this area is called the *modulus of elasticity* or *Young's modulus* that is a good measure of the stiffness of a material.  The Young´s modulus of the different groups of our study were calculated and statistically analyzed. Statistical average and standard deviation for each group was: Bard= 3.22 MPa±1.13; Ncm= 2.70 MPa±1.08; Phy= 4.09 MPa±2.23 and Vent=4.21MPa±1.64. 

Statistically significant differences were found (p<0.05) only when the groups Ncm and Vent were compared. 

## Discussion

Since abdominal wall repair using a prosthetic material has become routine clinical practice, several preclinical studies [24,25] have been designed to find the ideal prosthetic material in terms of its good behaviour at every interface. Initial composite designs [15] were composed of non-biodegradable polymer compounds, conditioning host tissue incorporation and behaviour at the peritoneal interface. Since their introduction, the composition of composites has changed to give rise to prostheses made of different absorbable materials. These were first used to replace the component designed to confer good peritoneal behaviour [26-28] and then used to make the component responsible for host tissue incorporation [20,29]. The idea of using biodegradable components is to leave behind as little foreign material in the host as possible, especially when a large mesh has to be used. These biomaterials should also achieve the regeneration of good quality tissue at the repair site in the host. Good clinical results in abdominal hernia surgery strongly depend on obtaining perfect correspondence between the mechanical properties of the abdominal wall and the mechanical properties of the biomaterial used for repair. Although as shown in some of our previous works, the regenerated tissue in some cases substantially modifies the original properties of the prosthesis [30,31]. Thus, once the mesh has adapted to the host tissue, the behaviour of the implant site should reproduce the behaviour of healthy tissue. 

The experimental model used in our study has been widely validated in prior studies conducted by our group [32]. The New Zealand White rabbit is a good model since it is of a size that can be easily handled, meaning that large abdominal wall defects can be created and repaired and laparoscopic monitoring is also possible. 

As main macroscopic complications, significant presence of seroma at the subcutaneous level was detected after the implant of *Vent*, followed by *Phy* and *Ncm*. This was never detected at long term, probably due to absorption of the biomaterial allowing the seroma to drain into the peritoneal cavity. However, occurrence of seroma in clinical practice is uncommon since aspirating drainage tubes prevent the build-up of fluid. 

The implants were conducted without overlapping and this gave rise also to a few button-hole hernias detected by laparoscopy at short term. The *Phy* implants gave rise to the highest hernia incidence, that could be attributed to the bilaminar structure, the partially reabsorbable mesh part and the very large pores that determines bad behaviour at the prosthesis/tissue interface. This type of herniation can occur in clinical practice making mesh overlap mandatory [33,34]. 

In our study, the intraperitoneal behaviour of the different composites was examined by sequential laparoscopy at the different time points. This gives additional value to our study. In agreement with others [35-37], laparoscopy has the benefit that the postimplant process can be followed in real time in a single animal making it possible to examine the peritoneal interface early after implant. Peak adhesion formation was observed at 7 days in the polypropylene controls with firm and integrated adhesions detected throughout the implant, in contrast with the different composites where adhesion formation was minimal. Others authors [18,20,24,29,38,39] have reported similar results for some of the composites examined here. We should highlight that adhesions had stabilized between days 7 and 14 for all composites. This observation, also mentioned by other authors [28,35], reveals that the critical moment for adhesion formation is within the first 7 days of implant [36,37]. In clinical studies [40-42] in which after the implant of a composite a laparoscopic revision surgery was needed for another condition, our finding of minimal adhesions was confirmed for prostheses of similar design to *Ncm*. 

For prostheses to become well integrated in the host tissue, collagen needs to become incorporated in the neoformed tissue. Collagen I is the main fibrillar protein responsible for the mechanical properties of tissue, hence our interest in correlating the presence of this protein with the results of our mechanical studies. Type III collagen is also a fibrillar collagen. It is the second most abundant collagen in human tissues and is found in extensible connective tissues and very common in fast growing tissue, particularly at the early stages of wound repair.

Host tissue incorporation into the prosthetic material was good in all our study groups. The different prostheses became fully infiltrated by a loose tissue after 14 days of implant which gained in density during the course of the study. This was reflected in our biomechanical studies where significantly higher tensile strengths were recorded at long versus short term. 

*Phy* showed the faintest collagen I protein expression while *Vent* showed the most intense expression at 14 days compared to the rest of the implants. In agreement with our study, other authors [20] have described less collagen deposition for Phy and argue that this confers the implants more flexibility, contributing to patient comfort. However, their surgical model was unlike ours without the creation of a wall defect and the implants were fixed on the peritoneal side to avoid any wound healing effects on the tissue incorporation of the meshes. 

Regarding gene expression, the neo-tissue developed between the prosthetic filaments showed a similar pattern of collagen 1 and 3 mRNA expression in *Bard mesh* and *Ncm*, with an expression peak for both types of collagen produced at 14 days that was significantly reduced by 90 days. This could indicate early transcriptional activation, which lead to more rapid, better quality tissue regeneration. *Phy* showed the opposite pattern showing increased expression at 90 days, which was significant for collagen 3. All the composites showed significantly higher collagen 1 mRNA levels compared to *Bard mesh* at long term. 

In summary, it can be stated that, compared to *Phy*, the *Ncm* mesh showed more rapid good quality tissue regeneration given that collagen 1 mRNA for Phy mesh was significantly lower than for Ncm at 14 days and Phy also showed the lowest collagen I protein expression in our immunohistochemistry study. This tissue regeneration could be favoured by the earlier barrier absorption and the three-dimensional macroporous structure of Ncm. Thus the entire process of protein synthesis seemed to be delayed in the Phy group. Nevertheless, in the long term this has no impact on the biomechanics of the implant area.

A reduced foreign body reaction induced by Phy relative to the response shown by other composites and macroporous meshes has been previously described [20] by other authors. Regarding the macrophage response to the implants, in our study Vent elicited the most intense response while the other two composites showed a similar response at each time point. This is a point against *Vent*. Probably this behaviour could be related to the large amount of foreign material that is forming part of this prosthesis. 

As revealed by our biomechanical tests, tensile strengths failed to vary among the composites, and increased over time in all cases. These findings correlated with the collagen I expression levels recorded for the different composites in the long term. The modifications made to the new composites should confer good tensile strength and abdominal wall compliance when the repair process is complete. However, the highest value of modulus of elasticity, at long term, was observed in Vent, that showed an increase statistically significant of the same respect to Ncm, which demonstrated the increase of stiffness of the area. Several authors have described that after the surgical implantation of an abdominal prosthesis, the repair process involving tissue formation on the area of the defect, provokes shrinkage and shortening of the mesh that is reflected its size reduction [43], being the main cause of discomfort and pain in the clinical patient. In our case, we have not observed tissue shrinkage in any of the implanted composites, but it should be taken into account, as some authors have already described, that the stiffness of the repaired tissue is modulated depending on the implanted prosthesis, so the abdominal tissue response is different depending on the prosthesis used [44].

In conclusion, our findings indicate that: a) behaviour at the peritoneal interface of all the composites tested, including the new composite mesh, was excellent in terms of good peritoneal regeneration and scarce adhesions formation; b) early after implant, a higher incidence of seroma and a more intense macrophage response were observed for the Ventralight^TM^ meshes; c) all three composites achieved appropriate collagen deposition translating to good biomechanical behaviour. The three-dimensional macroporous structure of the new composite mesh may induce the rapid regeneration of tissue within the mesh. 
